# Cushing's Syndrome in a Patient With Rathke's Cleft Cyst and ACTH Cell Hyperplasia Detected by ^11^C-Methionine PET Imaging—A Case Presentation

**DOI:** 10.3389/fendo.2020.00460

**Published:** 2020-07-22

**Authors:** Karol Piotr Sagan, Elzbieta Andrysiak-Mamos, Leszek Sagan, Przemysław Nowacki, Bogdan Małkowski, Anhelli Syrenicz

**Affiliations:** ^1^Department of Endocrinology, Metabolic and Internal Diseases, Pomeranian Medical University, Szczecin, Poland; ^2^Department of Neurosurgery, Pomeranian Medical University, Szczecin, Poland; ^3^Department of Neurology, Pomeranian Medical University, Szczecin, Poland; ^4^Department of Diagnostic Imagining, Collegium Medicum Nicolaus Copernicus University, Toruń, Poland

**Keywords:** cushing syndrome, Rathke's cleft cyst, methionine-PET, pituitary hyperplasia, case report

## Abstract

**Background:** Adrenocorticotropic Hormone (ACTH)-dependent Cushing's Syndrome (CS) is most often caused by a pituitary adenoma. Although rarely, it can also result from pituitary corticotroph cell hyperplasia (CH). Reports on concomitant pituitary lesions including ACTH-producing adenomas and Rathke's cleft cysts (RCCs) have been published. Positron emission tomography (PET), using ^11^C-labelled-methionine (MET) as a tracer and co-registered with magnetic resonance imaging (MRI) has been shown to be useful in the diagnosis of pituitary collision lesions, however, its role is still under investigation. In this work we present the case of a patient in whom CS was caused by non-adenomatous CH within the wall of an RCC.

**Case Summary:** In 2015 a patient with signs and symptoms of CS was referred to our Department. Biochemical studies repeatedly showed elevated midnight serum cortisol and ACTH levels. Magnetic resonance imaging of the sellar region revealed an RCC and MET-PET/MR showed heterogeneous labelled-methionine metabolism in the vicinity of the cyst's wall. Transsphenoidal surgery resulted in rapid, complete and lasting relief of symptoms. Histopathological examination demonstrated an RCC and CH.

**Conclusions:** Concomitance of pituitary focal lesions is a rare phenomenon. Methionine-labelled PET/MR may be useful in the diagnosis of collision sellar lesions, including CH. Corticotroph cell hyperplasia can present as mild and fluctuating hypercortisolaemia.

## Introduction

Rathke's cleft cyst (RCC) is a frequently encountered lesion of the sellar region, found in about 20% of autopsies (1). Symptomatic RCCs are, however, uncommon and only in rare settings require surgical treatment. Several studies have addressed the coexistence of pituitary focal lesions, referred to as collision lesions. Although a rare entity, the coexistence of RCCs and pituitary adenomas has gained interest in recent literature. Reports on concomitant ACTH adenomas with RCCs have been published indicating diagnostic difficulties in such cases. Occasionally, patients who undergo transsphenoidal surgery due to Cushing's Syndrome (CS) are diagnosed with corticotroph cell hyperplasia (CH). However, until now, no report on CH concomitant with an RCC has been published.

The diagnosis of CS has remained a challenge in clinical practice, with limited sensitivity and specificity of laboratory and imaging tests. For this reason clinical examination, and sometimes long-term observation, can be key to correct diagnosis. However, conflicting with this is the fact that early implementation of appropriate treatment can be invaluable, because this can prevent the development of numerous organ complications which can lead to increased mortality and reduced quality of life (2).

The diagnosis of coexistent pituitary focal lesions may be difficult based on magnetic resonance imaging (MRI) findings. Recently, positron emission tomography (PET), using ^11^C-labelled methionine as a tracer and co-registered with MRI (MET-PET/MR), has been reported to provide valuable information in the diagnosis of pituitary adenomas (3, 4). However, its role in clinical practice is still under investigation.

Animal models and *in vitro* studies have contributed to an understanding of the pathophysiological basis of corticotroph adenomas (CA) as well as their varying clinical picture. It is hypothesised that silent corticotroph adenomas (SCA) originate from the intermediate lobe, while adenomas causing full-blown CS originate from anterior lobal cells (5, 6). Some reports have indicated that patients with adenomas originating from intermediate lobe tissues may have mild symptoms of CS and differentiating these cases from ectopic Adrenocorticotropic Hormone (ACTH) secretion or pseudo-CS presents additional difficulty (7, 8).

In this work we present the case of a patient with CS caused by non-adenomatous ACTH cell hyperplasia within the wall of an RCC. Methionine-labelled PET/MR proved to be an important tool in the diagnosis and decisions concerning further treatment.

## Case Report

**Figure 1:**
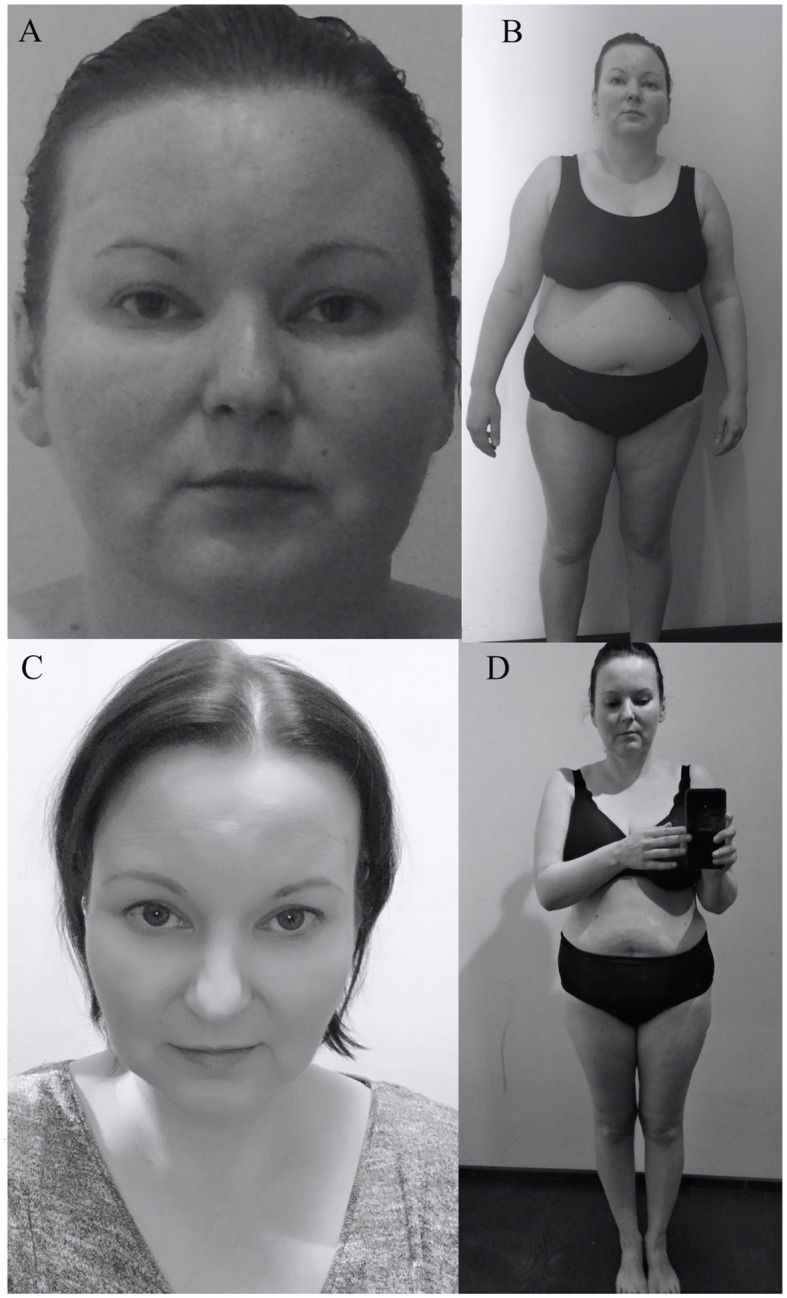
Photographs present the patient 3 months **(A,B)** and 36 months after surgery **(C,D)**.

A 35 years-old female patient, with previously diagnosed primary autoimmune hypothyroidism, came to our Endocrinology Outpatient Clinic in September 2015. At interview the patient reported an increase in body mass of around 30 kg over the past 5 years, lowered mood, decreased concentration, increased appetite, easy bruising, and insomnia. She also complained of proximal muscle weakness, which caused difficulty in climbing stairs to the first floor. Physical examination at that time revealed significant abdominal obesity (BMI 31.6 kg/m^2^), plethora, and dorsocervical fat pad (Figures 1A,B). The patient was not taking birth control pills and was not working shifts. In laboratory tests performed at the Endocrinology Outpatient Clinic in September 2015, abnormal findings were: leukocytosis with neutrophilia, elevated haemoglobin, hyperinsulinaemia, and elevated morning ACTH (72.47 pg/ml; normal level: 4.7–48.8); with cortisol levels near the upper limit (18.3 μg/dl; normal level: 6.2–19.4).

Due to the high pretest probability of CS the patient was hospitalised in our Department of Endocrinology in November 2015 to broaden diagnostics. In laboratory tests performed during the hospitalisation blood morphology was normal. Loss of the physiological circadian rhythm of cortisol secretion was diagnosed by the midnight serum cortisol measured on 2 consecutive days (cortisol level: 10.1 and 9.2 μg/dl; normal level: <7.5 μg/dl). Adrenocorticotropic Hormone levels were above the upper limit in the morning and at midnight (59.2 and 58 pg/ml). Correct inhibition of cortisol production was found in the 1 mg overnight dexamethasone suppression test (DST; cortisol level: 1.33 μg/dl) and urinary free cortisol level was within the normal range (101.78 μg/24 h; normal range: 36–137 μg/24 h).

**Figure 2:**
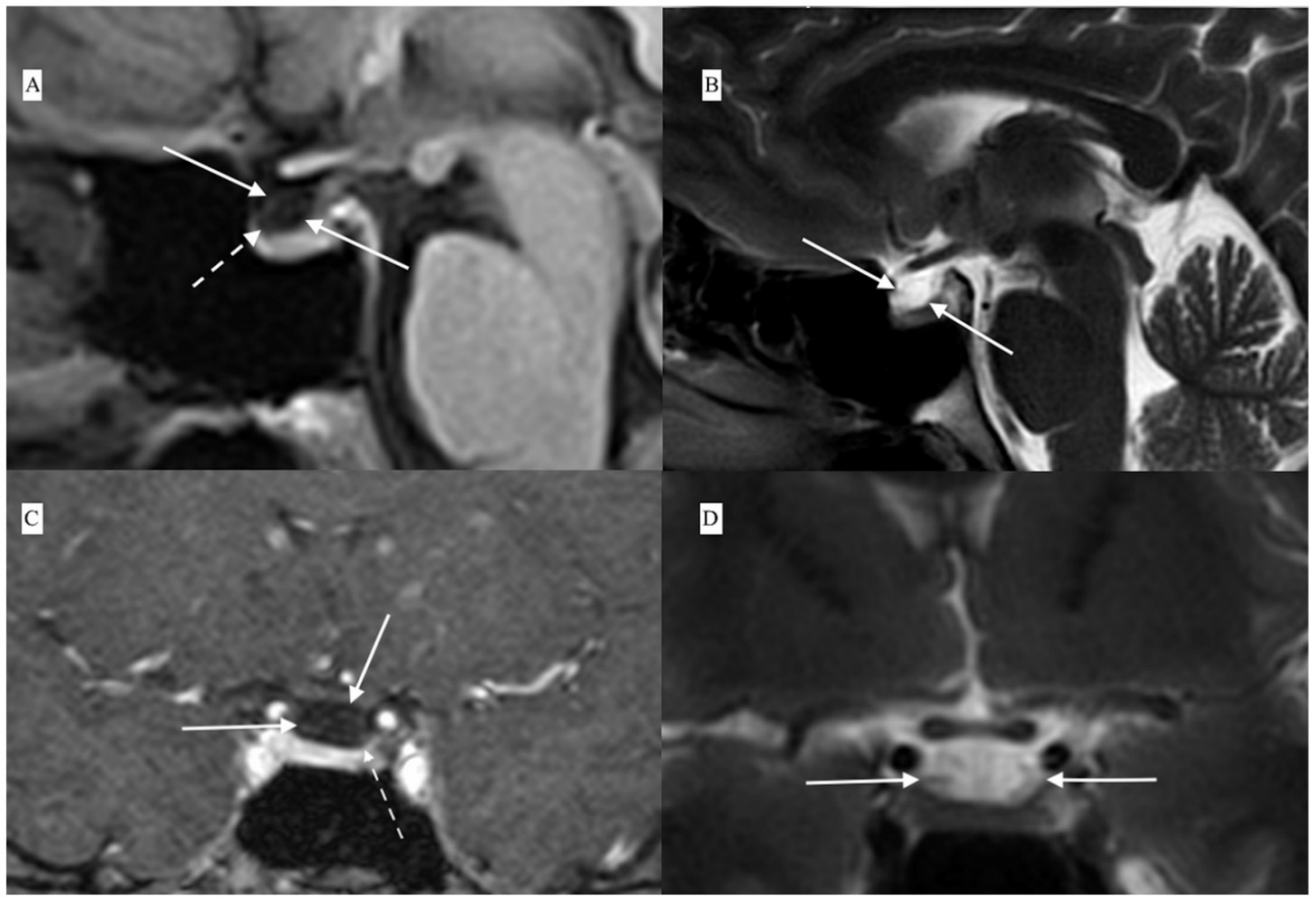
Contrast-enhanced MRI showing Rathke's cleft cyst (white solid arrows) anterior to the pituitary stalk and compressing the upper part of the pituitary: **(A)** T1-weighted contrast-enhanced sagital view, **(B)** T2-weighted sagital view, **(C)** T1-weighted contrast-enhanced coronal view, and **(D)** T2-weighted coronal view. Contrast enhancement of the lower part of the cyst wall in continuity with the pituitary tissue is visible in contrast-enhanced T1-weighted scans [dashed arrows in **(A,C)**].

**Figure 3:**
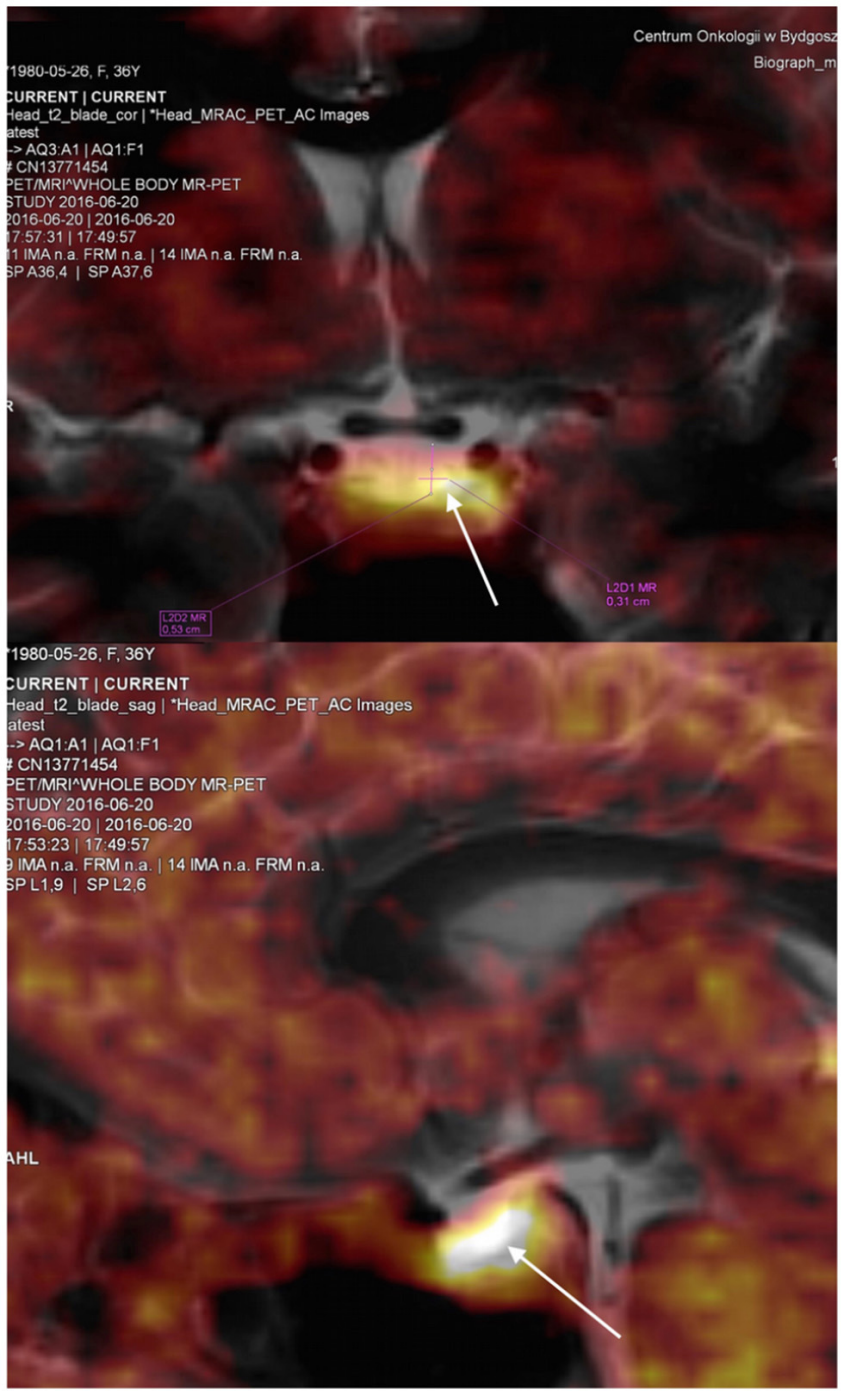
Coronal and sagital view of the pituitary visualised by positron emission tomography using ^11^C–methyl-L-methionine magnetic resonance imaging (MET-PET/MR). The study revealed the pituitary gland as being morfologically slightly thicker on the left side of the cyst with a peak of tracer uptake in this area. SUV max was 4.17 for this region compared to SUV max 3.14 in the remaining pituitary tissue. White arrows indicate the location of increased tracer uptake.

The findings indicated ACTH-dependent CS, possibly with intermittent variation in cortisol secretion. Magnetic resonance imaging of the pituitary gland, performed in May 2016, revealed a cystic lesion which was hypointense on contrast enhanced T1-weighted images and hyperintense on T2-weighted images and had typical features of RCCs (Figure 2). In June 2016, in a search for pituitary hyperfunction, MET-PET/MR examination was performed. The study was conducted using Biograph MR (Siemens) and ^11^C-methionine 20 min after injection of 720 MBq of the tracer. Time of acquisition was 15 min per bed. Positron emission tomography reconstruction was done using OSEM 3D. Magnetic Resonance sequences T2 Blade, T2 TSE were performed for the whole brain and T1 MPR, T1 TSE, T2 TSE sequences were performed for the sella. Slice thickness was 1–2 mm. The study showed a 6 × 3 mm cyst anterior to the pituitary stalk and the structure of the pituitary gland as being slightly thicker on the left side. Heterogenous ^11^C-methionine metabolism was observed around the cyst with a peak of tracer uptake on the left side of the cyst's wall (Figure 3).

Computed tomography (CT) of the chest and abdomen and scintigraphy of somatostatin receptors were performed. No pathology was found with these tests. The patient was rehospitalised in July 2016. Loss of the physiological circadian rhythm of cortisol secretion was again observed by high midnight serum cortisol level (10.6 μg/dl). Complete suppression of cortisol was again found in the low dose DST and urinary free cortisol measured twice was within normal limits. The other hormone levels were: Thyroid-Stimulating Hormone (TSH): 1.75 μIU/ml, L-Thyroxine (FT4): 1.26 ng/dl, Prolactin: 22.08 ng/ml, Follicle-Stimulating Hormone (FSH): 3.34 IU/ml, Luteinizing Hormone (LH): 1.45 mIU/ml, Growth Hormone (GH): 0.04 ng/ml (Table 1).

**Table 1:** Laboratory results.

	**09.2015 r**.	**11.2015**	**07.2016**	**02.2017**
Leu	12.75	6.18	9.69	8.67 (3.98–10)
(3.8–10) tys.ul				
Neu	8.67	-	-	-
(2.5–5.4) tys./ul				
Ery	5.13	4.75	4.77	3.76 (3.93–5.22)
(3.7–5.1) mln/ul				
Hgb	16.1	14.4	14.6	11.4 (11.2–15.7)
(12–16) g/dl				
PLT	202	255	301	174
(150–450) tys./ul				
Glucose 0'	81.4	79	76.2	80.8
(60–99) mg/dl				
Glucose 120'		86		
Insulin 0' uIU/ml	14.1	19.7		
Insulin 120' uIU/ml		112.9		
Cholesterol total mg/dl	197	193	182.1	
HDL Cholesterol mg/dl (>45)	52.5	42.2	40.9	
LDL (<115) Cholesterol	140.3	127.2	127.2	
TG (<150) mg/dl	68.9	182.9	110.6	
Na	142	141	139	146
(135–145) mmol/l				
*K*	4.25	4.41	4.5	4.83
(3.5–5.5)mmol/l				
Crea	-	0.84	0.73	0.8
mg/dl (0.5–0.9)				
TSH	2.65	1.75	1.58	<0.005
(0.27–4.2) uIU/ml	l-thyroxin supplementation	l-thyroxin supplementation	l-thyroxin supplementation	
FT4	1.37	1.09	1.26	1.32
(0.93–1.7) ng/dl	l-thyroxin supplementation	l-thyroxin supplementation	l-thyroxin supplementation	
FSH	-		3.34	0.51
(3.5–12.5)mIU/ml				
LH	-		1.45	<0.1
(2.4–12.6) mIU/ml				
Estradiol	-		150.3	<0.5
(12.5–166) pg/ml				
PRL	-	14.43	22.08	0.57
(6–29) ng/ml				
GH	-		0.04	-
(0.13–9.88) ng/ml				
IGF-1	-		163	77.9
(109–284)ng/ml				
ACTH	72.47	59.15	65.02	6.78
8:00 AM				
(4.7–48.8) pg/ml				
ACTH			69.13	
14:00 PM				
(4.7–48.8) pg/ml				
ACTH			59.66	
17:00 PM				
(4.7–48.8) pg/ml				
ACTH	-	58.3	51.27	3.64
midnight				
pg/ml				
Cortisol	18.33	14.54	15.13	0.64
8:00 AM				
(6.2–19.4) ug/dl				
Cortisol			19.25	
14:00 AM				
ug/dl				
Cortisol			10.36	
17:00 AM				
ug/dl				
Cortisol	-	10.1	10.58	0.81
midnight				
ug/dl				
Cortisol UFC	-	101.78	85.5; 86.2	99.05
				(on supplementation)
(36–137 ug/24 h)				
Cortisol in 1 mg dexamethasone test (ug/dl)	-	1.33	1.12	-

Column I—results of laboratory tests carried out at the Endocrinology Outpatient Clinic (September 2015).

Column II—results of laboratory tests performed before the operation during the first hospitalization (November 2015).

Column III—results of laboratory tests carried out during the second hospital stay (July 2016).

*Column IV—results of laboratory tests performed after the operation during hydrocortisone supplementation (February 2017)*.

During the next few months the patient's overall health status worsened. Due to problems with concentration and short term memory she was unable to continue work in a bank. The patient's body mass had increased by 3 kg despite attempts at a dietary regime (BMI 32.7 kg/m^2^). She developed stage 1 hypertension and treatment with ramipril was initiated. Proximal muscle weakness worsened and the patient was unable to hold a hairdryer sufficiently long to blow-dry her hair. After consideration of the whole clinical picture, and the results of the imaging tests, and after neurosurgical consultation, the decision was made to perform transsphenoidal pituitary resection. This surgery was performed in January 2017. During the operation, after opening of the sella, a cystic lesion in the upper part of the pituitary gland was exposed. The structure of the gland surrounding the cyst wall had a morphology resembling a pituitary adenoma.

**Figure 4:**
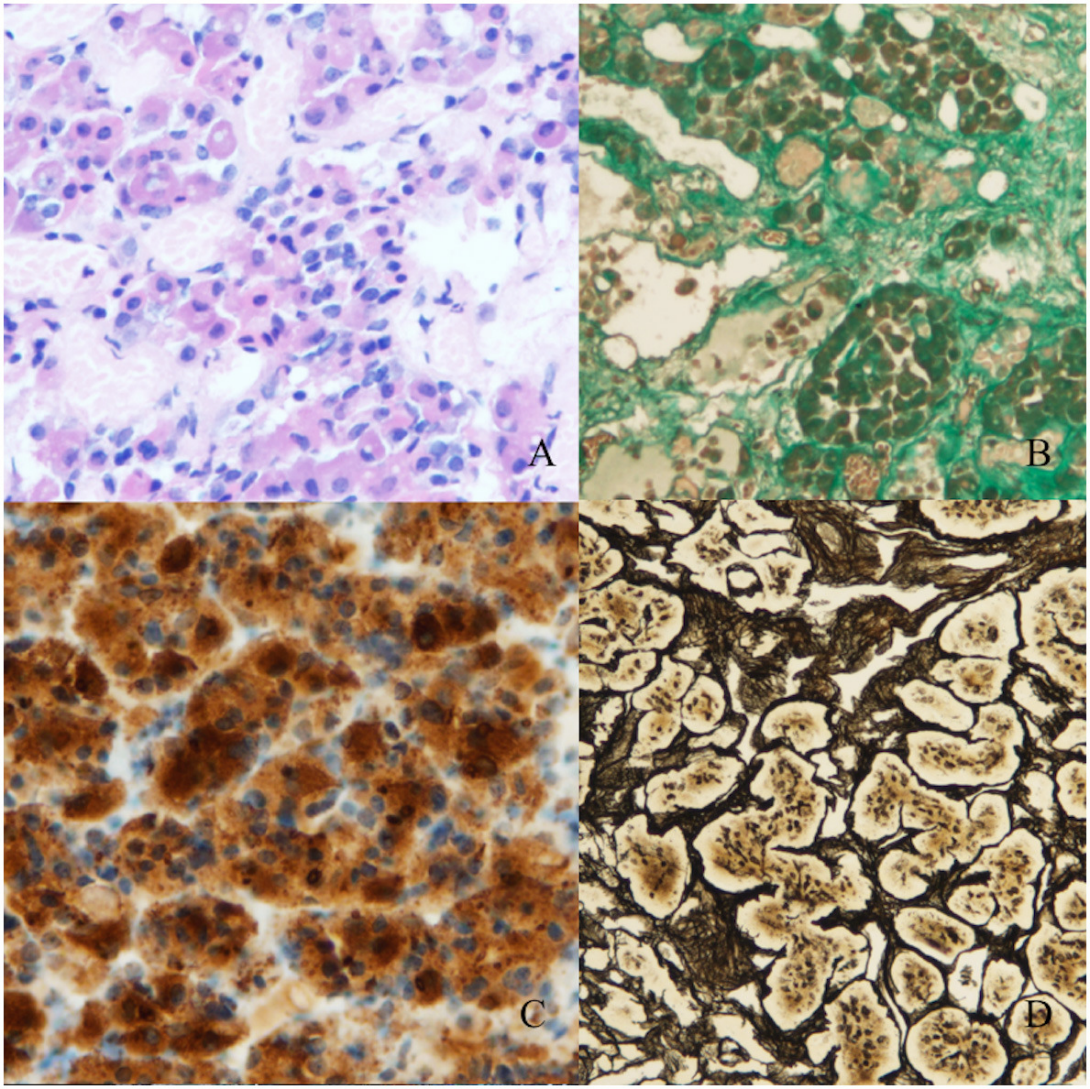
Histopathological material (magnification 100x, Olympus DP73). **(A)** Stained with hematoxylin and eosin. **(B)** Masson's trichrome staining for connective tissue. Numerous gland cell nests are visible between the fibres of connective tissue of the Rathke's cleft cyst. **(C)** Staining for ACTH. Numerous groups of ACTH+ cells are visible in the Rathke's cleft cyst's wall. **(D)** Reticulin staining shows a regular fiber pattern with numerous cell nests suggesting non-adenomatous cell hyperplasia.

Low ACTH (6.78 pg/ml) and cortisol (0.64 μg/ml) levels were found postoperatively. Secondary hypothyroidism, secondary hypogonadism and postoperative diabetes insipidus were diagnosed. Histopathological examination demonstrated an RCC, numerous ACTH+ cells and to a lesser extent TSH+ cells. Adrenocorticotropic Hormone positive cells had penetrated the connective tissue of the RCC. Reticular fibres retained a regular pattern indicating CH (Figure 4). The diagnosis was confirmed by two neuropathologists.

**Figure 5:**
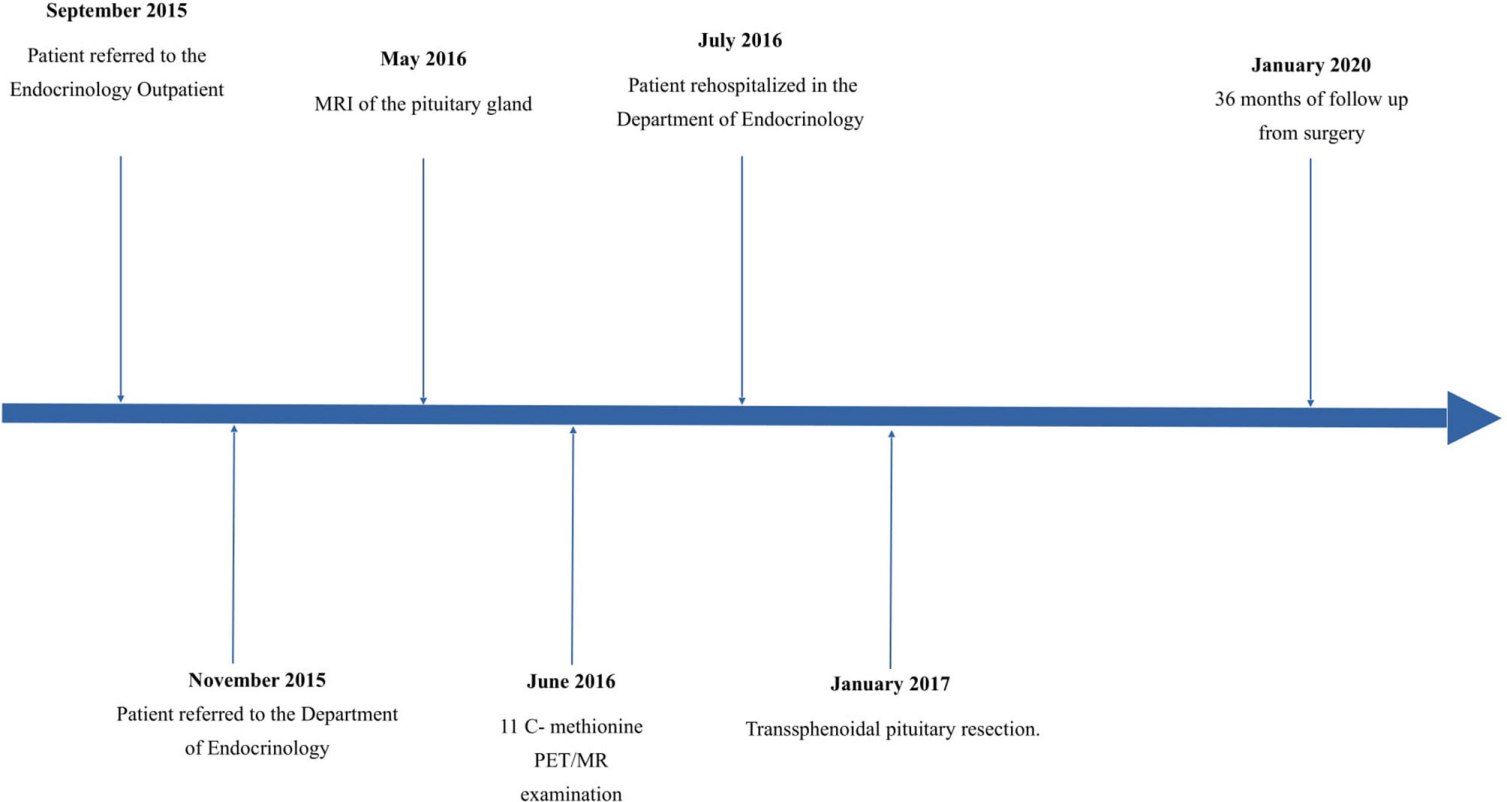
Timeline with information from the episode of care.

After the operation, the patient gained a significant and rapid improvement in mood and concentration and was able to resume work. Increased appetite, myopathy, hypertension resolved, and the plethora and dorsocervical fat pad disappeared. Over the next few months, a mass reduction of 21 kg was observed. Figure 1 presents the patient's photographs at 3 months (Figures 1A,B) and 36 months (Figures 1C,D) after surgery. Body composition measured by low-dosage, dual energy absorptiometry (DXA) technology (CoreScan™) before and after surgery revealed a reduction in total adipose tissue and truncal adipose tissue by 12.2 kg and 430 g, respectively. The patient was under the care of our Department for 36 months (Figure 5) and was aligned clinically and biochemically with replacement therapy: Hydrocortisone 10–5–0 mg, L-thyroxine 88/100 μg 1 × 1, dydrogesterone 10 mg (14 days a month) and estradiol 2 mg 1 × 1.

## Discussion

The presented case provides an example of the coexistence of two focal lesions in the pituitary and addresses the diagnostic difficulties concerning this rare entity. To our knowledge, it is the first description of CH concomitant with an RCC. Additionally, the authors did not find any other description of the use of MET-PET/MR in diagnosing CH in humans.

Collision lesions in the pituitary have previously been observed and knowledge about this phenomenon is primarily based on case reports, which may suggest an element of randomness. The challenge in diagnosing concomitant lesions results from the fact that an adenoma, located in a pituitary compressed by a cyst, may be undetectable by conventional MRI (9). In fact, inflammation has been found in 50% of RCC cases (10) and autopsies have revealed the presence of cyst wall metaplasia in 9–30% of RCCs (11). Whether metaplasia results in the development of adenoma around the cystic lesion remains controversial. In a prospective study by Ikeda et al. (12) pituitary adenomas were found to coexist with 34% of ruptured RCCs. A high percentage of these adenomas were diagnosed using MET-PET/MR and in this case series ACTH-producing adenomas accounted for one third of the concomitant adenomas. However, in histopathological studies, RCCs have been found in only 0.5–1.9% cases of pituitary adenomas (13–18). In the study by Nishio et al., RCCs were associated with 1.9% of pituitary adenomas in 464 patients (19) and in a recent retrospective study of 554 resected pituitary adenomas, 0.9% had a concomitant RCC (13). In a review of the literature including 32 cases of an RCC concomitant with pituitary adenoma Noh et al. identified only two cases of ACTH-producing adenomas causing CS (17). Recently, however, another six patients with ACTH-producing adenoma concomitant with an RCC have been reported (16, 20–23).

Methionine is an aminoacid, which is incorporated into all aminopeptides. Labelling methionine with ^11^C is therefore a promising tool in localising regions with increased peptide synthesis, including pituitary adenomas. Studies have shown a very high sensitivity of this method in localising these tumours (24–27). In a series of patients with a recurrent pituitary adenoma, MET-PET/MR improved gamma-knife radiosurgery-targeting and increased the proportion of patients considered for this treatment (28). Koulouri et al. has shown the utility of MET-PET/MR in a series of patients with *de novo* diagnosed, residual or recurrent ACTH-secreting tumours (29). Additionally, Tobin et al. has proposed that this method can aid localisation of ACTH-dependent CS when conventional imaging is inconclusive (30).

For comparison, Tomura et al. presented measurements of ^11^C-methionine uptake by probably normal pituitary gland (31). In these cases tracer uptake was uniform. Importantly, the study used PET/CT technology, while in our case we used PET/MR. The tracer uptake measured using maximum standardized uptake values (SUV max) by PET/MR may be different from that obtained with PET/CT. In the study by Lyons et al., PET/MR compared with PET/CT underestimated SUV (32). In our patient no focal lesion was seen with MET-PET/MR, but asymmetric ^11^C-methionine metabolism suggested pathology on the left side of the gland (Figure 3), confirmed during neurosurgery.

Occasionally ACTH-dependent CS is caused by non-adenomatous pituitary CH (33–43). This pathology is characterized by expanded ACTH-positive cells with a preserved pattern of reticular fibres. Pathomorphological studies have shown that non-adenomatous cell proliferation can precede adenoma formation (44). According to one hypothesis there is some continuity between non-focal cell proliferation and a subsequent focal lesion. Mazarkis et al. (45) reported a unique case of somatotroph adenoma combined with ACTH-immunoreactive cell hyperplasia with focal transformation to adenoma. A similar case was reported by Haap et al. (46).

Although CH is perceived as a rare cause of CD, Castlen et al. (47) has shown that it accounted for 13 of 104 corticotroph lesions removed by transsphenoidal surgery. In their study patients with CH did not differ from those with CAs in terms of cortisol and ACTH levels but more often exhibited mood changes and cognitive dysfunction and had more discrete changes identified in pre-operative MRI. Moreover, these patients presented with a greater number of negative late-night salivary cortisol results. This phenomenon can be most likely explained by episodic or cyclical cortisol production in these patients. In line with this view, Noctor et al. presented a case of a 13-years old boy with CH with cyclical Cushing's disease (48).

By definition cyclical CS is diagnosed by showing three peaks and two troughs of cortisol with similar time distances between the peaks (49). These criteria may not be achieved in all patients as the intercyclic interval can be long (50). The study by Alexandraki et al. suggested the diagnosis of cyclical CS by the existence of only one cycle, expressed as two peaks with one trough of hypercortisolaemia (51). Cases in which excess cortisol production occurred in cycles lasting from 12 h to years have been described in the literature. Episodic CS refers to elevated cortisol levels occurring with no temporal pattern (52). This phenomenon seems to occur more often than has been previously thought and has presumably been overlooked. There is therefore growing evidence that fluctuations in cortisol secretion occur in patients who do not meet the criteria for cyclical CS (53). Meinardi et al. suggested that cyclical/episodic cortisol production is present in at least 15% of patients with CD (50). In their study, Friedman et al. presented, that episodic CS may be common and simultaneously revealed that current screening tests performed once are inadequate to detect or exclude hypercortisolism in patients with mild or episodic CS (52). Interestingly, in their case series, one patient with CD presented only with repeatedly elevated midnight cortisol levels as was shown in our presented case. Moreover, the authors emphasised that an ACTH-adenoma is often invisible in postoperative material.

Several reports have suggested that subclinical or cyclical hypercortisolaemia is typical for tumours which originate in middle-lobe tissues, due to lower concentrations of pro-opiomelanocortin convertase 1/3 in these tumours than in anterior lobal cells (54). Possibly the ACTH produced by these cells might have an unusual structure: although it is detected by commercially-available antibodies, apparently it has weaker affinity for the receptor in the adrenal glands, resulting in weaker cortisol secretion (5). It is also hypothesised that silent corticotroph adenomas (SCA) originate from the intermediate lobe. In our patient hyperplasia was found around the cyst wall of the RCC, most likely from cells originating from the intermediate lobe.

The difficulty in diagnosing CS in the present case was due to quite mild clinical hypercortisolaemia with normal response to the 1 mg overnight DST and normal cortisol concentration in 24 h urine collection. Importantly, a high awake midnight serum cortisol level as well as a lack of ACTH diurnal rhythm were repeatedly noted. According to the Endocrine Society's Clinical Practice Guidelines: a midnight serum cortisol level >8.3–12 μg/dl has a specificity of 96% for CS (55). Moreover, some authors have suggested that a 1 mg overnight DST may give a false negative in mild cases of CS (56). In patients with mild or cyclical hypercortisoliaemia, urinary free cortisol may also be normal (57). In line with this, Raff et al. conclude that “free cortisol only appears in the urine when its concentration exceeds the binding capacity of plasma” and “a patient with a serum cortisol of 12 μg/dl all day long is likely to have Cushing's syndrome but might have a normal 24-h UFC measurement” (58). It seems that our patient presented this clinical scenario. One explanation is fluctuating cortisol overproduction as discussed above. Another possible explanation is that midnight serum cortisol level is the most sensitive test in diagnosing early CD (59). This test has been proposed in defining initial remission after surgical treatment of pituitary ACTH-producing adenomas (60, 61). The diagnosis in our case was supported by strong clinical evidence.

Mild ACTH-dependent Cushing's syndrome should also be differentiated from pseudo-Cushing's syndrome. The patient did not have underlying disease that could be a cause of pseudo-Cushing's syndrome (e.g., depression, alcoholism, uncontrolled diabetes, or sleep apnea syndrome). In addition, as reported by Alwani et al. (62), high evening cortisol concentrations with a midnight:morning serum cortisol ratio over 0.67 as well as observation of the patient for several months spoke against this diagnosis. Corticotroph cell hyperplasia can also arise due to secondary ectopic corticotropin-releasing hormone (CRH), however it is unlikely in this case as the patient went into remission after pituitary surgery. Complete and lasting relief of the symptoms of CS was confirmed by long term follow-up.

Thyroid-Stimulating Hormone positive cell proliferation in our patient, although found to a significantly lesser degree compared to ACTH+ cell proliferation, remains difficult to explain. As mentioned above, the patient also suffered from primary hypothyroidism, so the proliferation of this cell line could have been a physiological response to hypothyroidism. Another explanation is that the resected pathological lesion was plurihormonal. Immunochemistry for transcription factors is currently unavailable in our centre and was not performed.

The authors are aware of the limitations of the study. Inferior Petrosal Sinus Sampling (IPSS), which is a gold standard in differentiating ectopic from central CS, was not performed as it is not available in our centre. However, studies have shown that IPSS may give false negative results in cases of cyclical CD (40, 48, 63, 64). Nishioka et al. suggest that with the development of functional imaging studies the significance of the procedure may lessen (65), although this view remains unproven. In our case, in the search for an ectopic source of ACTH, receptor scintigraphy and chest and abdomen CT were performed. Pituitary hyperfunction was assessed with the use of MET-PET/MR.

Cushing's Syndrome still remains an underdiagnosed disease (66). As RCCs rarely cause symptoms, in most cases their treatment does not require surgical intervention and verification of diagnoses is therefore rarely possible. Early detection of pathological pituitary-cell proliferation could prevent numerous organ complications and reduce operational risk.

Methionine-labelled PET/MR may prove useful in detecting pituitary-cell proliferation and the usefulness of PET in detecting hyperplasia has recently been demonstrated in an animal model by Balcerzyk et al. (67). However, the usefulness of MET-PET/MR in the differential diagnosis of mild hypercortisolaemia with CH from pseudo-CS requires further research (68).

## Conclusions

Concomitance of pituitary focal lesions is a rare phenomenon. Methionine-labelled PET/MR may be useful in the diagnosis of collision sellar lesions, including CH. Corticotroph cell hyperplasia can present as mild and fluctuating hypercortisolaemia.

## Data Availability Statement

The datasets generated for this study are available on request to the corresponding author.

## Ethics Statement

Ethical review and approval was not required for the study on human participants in accordance with the local legislation and institutional requirements. The patients/participants provided their written informed consent to participate in this study. Written informed consent was obtained from the individual(s) for the publication of any potentially identifiable images or data included in this article.

## Author Contributions

KS: collected data, performed literature search, and drafted the manuscript. EA-M, KS, LS, BM, and AS: performed medical and surgical procedures. AS: made critical revision and contributed to the writing of the manuscript. PN: prepared histopathological results. All authors revised, approved the final manuscript, and agreed to be accountable for the content of the work.

## Conflict of Interest

The authors declare that the research was conducted in the absence of any commercial or financial relationships that could be construed as a potential conflict of interest.
